# Comparison of the Phytochemical Properties, Antioxidant Activity and Cytotoxic Effect on HepG2 Cells in Mongolian and Taiwanese Rhubarb Species

**DOI:** 10.3390/molecules26051217

**Published:** 2021-02-25

**Authors:** Ganbolor Jargalsaikhan, Jin-Yi Wu, Yen-Chou Chen, Ling-Ling Yang, Ming-Shun Wu

**Affiliations:** 1 International MS/PhD Program in Medicine, College of Medicine, Taipei Medical University, Taipei 11031, Taiwan; d142107013@tmu.edu.tw (G.J.); yc3270@tmu.edu.tw (Y.-C.C.); 2 Liver Center, Ulaanbaatar 14230, Mongolia; 3 Department of Microbiology, Immunology and Biopharmaceuticals, College of Life Sciences, National Chiayi University, Chiayi 60004, Taiwan; jywu@mail.ncyu.edu.tw; 4 Graduate Institute of Medical Sciences, College of Medicine, Taipei Medical University, Taipei 11031, Taiwan; 5 Cancer Research Center and Orthopedics Research Center, Taipei Medical University, Taipei 11031, Taiwan; 6 Cell Physiology and Molecular Image Research Center, Wan Fang Hospital, Taipei Medical University, Taipei 11031, Taiwan; 7 School of Pharmacy, College of Pharmacy, Taipei Medical University, Taipei 11031, Taiwan; llyang@tmu.edu.tw; 8 American College of Acupuncture and Oriental Medicine, Houston, TX 77063, USA; 9 Division of Gastroenterology, Department of Internal Medicine, Wan Fang Hospital, Taipei Medical University, Taipei 11031, Taiwan; 10 Division of Gastroenterology and Hepatology, Department of Internal Medicine, School of Medicine, College of Medicine, Taipei Medical University, Taipei 11031, Taiwan; 11 Integrative Therapy Center for Gastroenterological Cancers, Wan Fang Hospital, Taipei Medical University, Taipei 11031, Taiwan

**Keywords:** *Rheum undulatum* L., *Rumex crispus* L., Rhubarb, phytochemicals, antioxidant, liver cancer

## Abstract

The Mongolian rhubarb—*Rheum undulatum* L. *(RU)*—and *Rumex crispus* L. *(RC)*—a Taiwanese local rhubarb belonging to the family of *Polygonaceae*—are principal therapeutic materials in integrative medicine due to their rich quantities of bioactive compounds; however, their phytochemical and antioxidant properties, and anti-cancer activity is poorly investigated. Furthermore, the phytochemical characteristics of both species may be affected by their different geographical distribution and climatic variance. The current study aimed to compare *RU* with *RC* extracts in different polarity solvents (n-hexane, ethyl acetate, acetone, ethanol, and water) for their phytochemical contents including the total phenolic content (TPC), total anthraquinone content (TAC), total flavonoid content (TFC), antioxidant and free radical scavenging capacities, and anticancer ability on the HepG2 cell. Except for the n-hexane extract, all of the *RU* extracts had considerably higher TPCs than *RC* extracts, ranging from 8.39 to 11.16 mg gallic acid equivalent (GAE) per gram of dry weight, and the TPCs of each extract were also significantly correlated with their antioxidant capacities by ABTS, DPPH, and FRAP assays (*p* < 0.05). Moreover, there was no remarkable association between the antioxidant capacities and either TACs or TFCs in both the *RU* and *RC* extracts. Besides, high-performance liquid chromatography (HPLC) analysis revealed that both the *RU* and *RC* extracts contained chrysophanol, emodin, and physcion, and those bioactive compounds were relatively higher in the n-hexane solvent extracts. Additionally, we observed different levels of dose-dependent cytotoxic effects in all the extracts by cell viability assay. Notably, the ethanol extract of *RU* had a compelling cytotoxic effect with the lowest half-maximum inhibition concentration (IC50-171.94 ± 6.56 µg/mL at 48 h) among the *RU* extracts than the ethanol extract of *RC*. Interestingly, the ethanol extract of *RU* but not *RC* significantly induced apoptosis in the human liver cancer cell line, HepG2, with a distinct pattern in caspase-3 activation, resulting in increased PARP cleavage and DNA damage. In summary, Mongolian Rhubarb, *RU*, showed more phytochemical contents, as well as a higher antioxidant capacity and apoptotic effect to HepG2 than RC; thus, it can be exploited for the proper source of natural antioxidants and liver cancer treatment in further investigation.

## 1. Introduction

The Rhubarb (da huang) is a well-known herb that has been used as a vegetable as well as an essential constituent in traditional medicine for more than 2000 years. Researchers have broadly studied *Rheum palmatum, Rheum officinale*, and *Rheum tanguticum*, called official rhubarb, rather than the unofficial rhubarb species such as *Rheum undulatum* L. (*RU)* and *Rumex crispus* L. *(RC)* [1,2]. *RU*, cultivated in some countries of East Asia and Eastern Europe such as Mongolia, Korea, and Russia, has been used in integrative medicine due to its rich source of bioactive contents [3]. *RU* extract has been studied for its therapeutic effects in the treatment of various diseases, and for its identified active compounds, namely emodin, aloe-emodin, chrysophanol, rhein, and stilbene [4,5,6,7], but *RU* from Mongolia has not been studied. *RC*, locally known in Taiwan as da huang, is an endemic plant in many countries of Eurasia that particularly tends to grow in humid weather [8]. In ancient times, *RC* was effectively used in the treatment of gastrointestinal disorders, skin diseases, and urinary tract infections [9]. Studies from Russia and Korea have thoroughly explored the phytochemical contents and antioxidant, anti-inflammatory, and anticancer capacities of *RC* extract in different solvents, and its active compounds such as chrysophanol, emodin, and physcion [10,11].

Hepatocellular carcinoma (HCC), accounting for >80% of primary liver cancers, is a leading cause of cancer death in many parts of the world due to its late-stage diagnosis, a lack of effective treatment, and serious adverse events of approved drugs [12]. Mongolia has the highest prevalence of HCC in the world, with a prevalence that is 10 times higher than the world average, and HCC has been the second most common cause of mortality after cardiovascular diseases in Mongolia since 2002 [13,14]. In contrary, Taiwan has the highest survival rate of HCC in the world, but HCC is still the leading reason of cancer-caused death in Taiwan [15]. As long as new curative treatment is compulsory for HCC, developing drugs from herbal medicine could find optimal treatment options. Natural products such as medicinal herbs and their phytochemicals such as phenolic, flavonoid, and anthraquinone have been well-studied for their therapeutic and preventive effect on liver diseases such as liver cancer due to their readily available source, less adverse effects, successful treatment outcome, and cost-effectiveness [16].

Moreover, numerous studies have demonstrated that differences in the geographical area and climatic change significantly affect plant constituents, which means the same plant species in different regions may not yield identical compounds or the same amounts of constituents [17,18]. Thus, this study was conducted to estimate the phytochemical contents and antioxidant properties of Mongolian rhubarb extracts, and to study its anti-liver cancer ability in comparison with Taiwanese rhubarb extracts.

## 2. Results

### 2.1. Total Phenolic, Anthraquinone, and Flavonoid Contents

We screened for the presence of phytochemical contents by performing qualitative analyses and then estimated the total phenolic content (TPC), total flavonoid content (TFC), and total anthraquinone content (TAC) in both *RU* and *RC* extracts according to the specified protocols (Table 1). As shown in Table 1, except for the n-hexane (n-Hex) extract of *RU* (2.44 ± 0.69 mg GAE/g DW), all *RU* extracts had considerably higher TPCs than those of *RC* extracts (*p* < 0.001). Among the *RU* extracts, ethanol (EtOH) and ethyl acetate (EtOAc) extracts exhibited comparatively higher phenolic contents than other solvents. By contrast, no significant difference was observed in the TPCs of *RC* extracts in different solvents (*p* > 0.05), while, the n-Hex extracts of both *RU* and *RC* species exhibited the highest concentrations of TAC compared with other samples (*p* < 0.0001). Furthermore, the TAC of the n-Hex extract of *RU* was higher than that of *RC* (*p* < 0.05). The TFCs of the n-Hex extract followed by the acetone (Ac) and EtOAc extracts of *RC* were higher than those of the same solvent extracts of *RU* (*p* < 0.05). Solvent-based differences in TFC were not observed in *RU* extracts (*p* > 0.05).

### 2.2. Determination and Quantification of Bioactive Constituents of Each Extract through High-Performance Liquid Chromatography (HPLC) Assay

**Figure 1:**
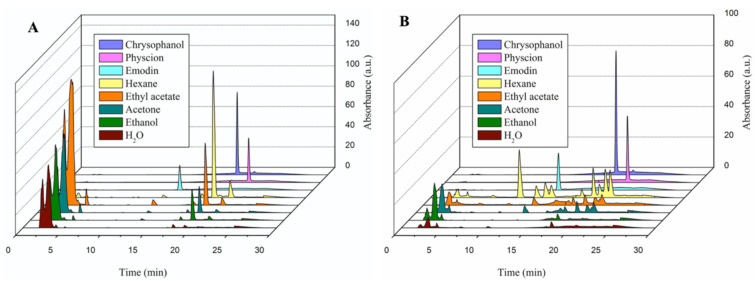
Identification and quantification of the bioactive constituents of *Rheum undulatum* L. and *Rumex crispus* L. through high-performance liquid chromatography. The HPLC spectrum of the bioactive constituents of *Rheum undulatum* L. (*RU*) (**A**) and *Rumex crispus* L. (*RC*) (**B**) extracts in different solvents. Chrysophanol (purple), physcion (pink), and emodin (light blue) were reference compounds. Each color represents different extract of *RU* and *RC*.

The bioactive compounds in *RU* and *RC* extracts were identified using HPLC analysis. Emodin, physcion, and chrysophanol were used as reference compounds, and the results of these extracts are displayed in Figure 1. HPLC chromatograms revealed the presence of the aforementioned three compounds in all of the *RU* and *RC* extracts (Figure 1A,B).

The concentrations of the three bioactive compounds, which differed among the extracts, are summarized in Table 2. Reference compounds had higher concentrations in *RU* extracts than in *RC* extracts. Water extracts of both *RU* and *RC* exhibited the lowest value of the reference compounds. For both *RU* and *RC*, n-Hex extracts exhibited the highest chrysophanol and physcion contents compared with other extracts. A higher quantity of emodin was noted in the EtOAc and n-Hex extracts of *RU* and *RC* than in any other extracts.

### 2.3. Antioxidant Activities of Each Extract

We evaluated the antioxidant and free radical scavenging activities of *RU* and *RC* extracts by using ABTS, DPPH, and FRAP assays. For the ABTS and DPPH assays, the positive controls were gallic acid (GA) and Trolox. The results of ABTS and DPPH scavenging assays were expressed as the half-maximum inhibition concentration to reduce free radicals (Table 3). The FRAP assay was used to measure the reduction of Fe^3+^ to Fe^2+^, with a high value for Fe^2+^ indicating superior antioxidant ability. Antioxidant properties were observed in all extracts to different extents. Of all the extracts, the EtOAc, Ac, EtOH, and water extracts of *RU*, which had high TPCs, exhibited high antioxidant activity with the lowest inhibition concentration compared with the same extracts of *RC* (*p* < 0.0001). All of the free radical scavenging assays showed that the n-Hex extract of *RC* had a greater inhibition ability than that of *RU* extract in n-Hex.

### 2.4. Cytotoxicity of RU and RC Extracts on Liver Cancer Cell Lines

To estimate the anti-liver cancer effects of the extracts, an MTT assay was performed on HepG2 cell lines treated with various concentrations of extracts at three time points (24, 48, and 72 h) (Table 4). Table 4 shows that the cytotoxic effects of the *RU* extracts were significantly higher than those of the *RC* extracts at all time points. Owing to the excessive concentrations of *RC* extracts used to inhibit HepG2 cell growth, we could not obtain the cytotoxic doses of *RC* in n-Hex, EtOAc, and Ac solvents at the 24-h time point. The time-dependent cytotoxic effects of *RU* extracts were significantly different at the 24-h time period compared with at 48 and 72 h (24 h vs. 48 h; 24 h vs. 72 h, *p* < 0.05). In the case of *RC* extracts, the n-Hex and EtOAc extracts exhibited strong time-dependent cytotoxic effects (48 h vs. 72 h; *p* < 0.05). The time point at which the cell growth of all extracts was significantly inhibited was 48 h. Notably, the EtOH extract of *RU* (*RU*-EtOH) had compelling cytotoxic effects, with the lowest IC50 values at all time points (262.28 ± 13.99; 171.94 ± 6.56; and 167.09 ± 8.99 µg/mL; *p* < 0.05).

### 2.5. Elucidation of Liver Cancer Cell Death

**Figure 2:**
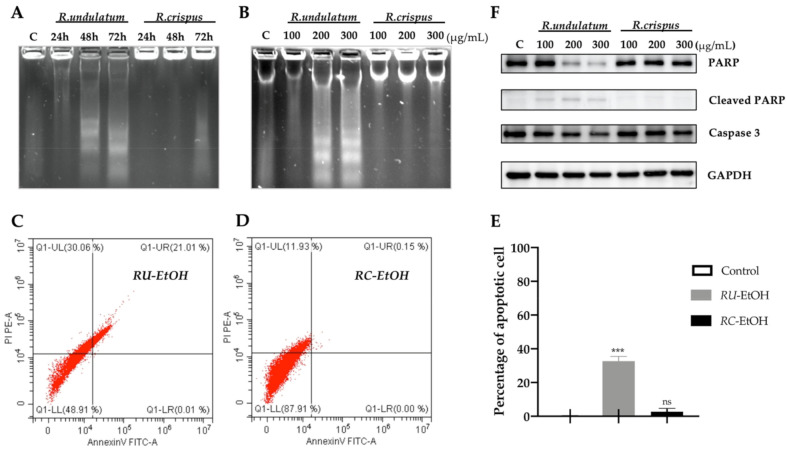
*RU*, not *RC*, induces significant apoptosis responses in human hepatocellular carcinoma cell line HepG2. DNA fragmentation using agarose gel electrophoresis of DNA isolated from HepG2 cells treated with either *RU*-EtOH or *RC*-EtOH (**A**,**B**). Annexin V-FITC/PI flow cytometric shape of HepG2 cells, 48 h post-treatment of the ethanol extracts of *RU* (**C**) and *RC* (**D**). Compared with negative control (DMSO), apoptotic percentage of the total cell population was presented and given as mean ± SD (*n* = 3) *** *p* < 0.0001; ns: not significant (**E**). Effects of different concentrations of *RU*-EtOH and *RC*-EtOH on the protein expression responsible for apoptosis in HepG2 cells. GAPDH was used as an internal control (**F**).

After the *RU*-EtOH was found to have a more potent cytotoxic effect on HepG2 cells than that of other extracts, further experiments were conducted using the EtOH extracts of *RU* and *RC*. The constituents of *RU* and *RC* extracts identified through HPLC analysis in this study are known to induce cell death through different pathways [19,20,21]. Therefore, we attempted to determine which cell-death type is affected by the EtOH extracts of *RU* and *RC*. We performed a DNA fragmentation assay to detect the apoptotic effect of the extracts. The results are displayed in Figure 2. Compared with the EtOH extract of *RC* (*RC*-EtOH), the EtOH extract of *RU* (*RU*-EtOH) had a stronger stimulating effect on the loss of DNA integrity and DNA ladder formation, and this effect was dose dependent as well (Figure 2A,B). DNA ladders were observed at both 48 and 72 h after treatment incubation of *RU*-EtOH (Figure 2A). However, there was no significant difference between the results at 48 and 72 h.

Then, the HepG2 cells were treated with the same concentrations of *RU*-EtOH and *RC*-EtOH extracts for 48 h, and the percentage of apoptotic cells was measured through double staining (annexin V-FITC) and flowcytometry (CytoFlex, Beckman Coulter, Indianapolis, Indiana, USA). The results shown in Figure 2C indicate that that the cell distribution pattern was different in *RU*-EtOH and *RC*-EtOH exposed HepG2 cells. The apoptotic cells were observed more when *RU*-EtOH was used for treatment than when *RC*-EtOH was used (21.02% vs. 0.15%; Figure 2C).

PARP and caspase-3 are responsible proteins in the apoptotic program. HepG2 cells were exposed to different doses of *RU* and *RC* extracts in EtOH for 48 h, and the expressions of PARP, cleaved PARP, and caspase-3 were evaluated through western blotting (Figure 2D). *RU*-EtOH diminished the expression of PARP and caspase-3 proteins and increased the level of cleaved PARP compared with those of *RC*-EtOH, and these effects were dose dependent. These data, which agree with other cell experiment results, confirm that *RU*-EtOH induces more apoptosis than *RC*-EtOH does in HepG2 cells, with a characteristic pattern in annexin V-FITC, activation of caspase-3, and cleavage of PARP.

All the results suggest that some of the anticancer mechanisms in the ethanol extract of *RU* are attributable to apoptosis in the HepG2 cells.

## 3. Discussion

To the best of our knowledge, this is the first study to describe the phytochemical content, antioxidant free radical scavenging activity, and anti–liver cancer effects of *RU* from Mongolia. In addition, we compared two unofficial rhubarb species (da huang) cultivated from two different areas—Mongolia and Taiwan—and extracted them in five different polarity solvents (n-Hex, EtOAc, Ac, EtOH, and water). The results of this study revealed that *RU* extracts (Mongolian rhubarb) had higher TPCs, TFCs, and TACs, superior antioxidant abilities, and greater induced apoptosis of liver cancer cells than did *RC* extracts (Taiwanese rhubarb). The TPCs of the *RU* and *RC* extracts were significantly associated with antioxidant capabilities measured using ABTS, DPPH, and FRAP assays (*p* < 0.05), which supports previous findings [22]. However, the antioxidant capabilities of the *RU* and *RC* extracts were not associated with their flavonoid and anthraquinone contents (*p* > 0.05).

Trinh stated that aloe-emodin, chrysophanol, rhapontigenin, and rhaponticin were isolated from the *RU* extracts and their fractions [23]. Nevertheless, our study identified chrysophanol, emodin, and physcion to be the major compounds in the *RU* extracts. The concentrations of these compounds were particularly high in n-Hex, EtOAc, Ac, and EtOH solvents. Similar to previous studies [10,24], our study found chrysophanol, emodin, and physcion in the *RC* extracts. Furthermore, a comparable trend has documented that the water extract of *RC* showed the lowest value of pure compounds [24]. From these results, which are consistent with those of a study by Lee [25], it is clear that pure compounds are extracted mostly in nonpolar and moderately polar solvents, such as n-Hex, EtOAc, and EtOH.

As we mentioned above, antioxidant activities of *RU* and *RC* extracts significantly correlated with phenolic contents, but not with flavonoid and anthraquinone contents. Moreover, there was no correlation between pure compound concentrations and antioxidant activities. It can be explained that there might be some synergistic and antagonist effects between bioactive compounds in *RU* and *RC* extracts according to the previous report [26,27].

Previous studies have investigated official rhubarb species, such as *R.palmatum*, and mostly studied their active compounds and their anti-liver cancer activities [2,28,29]. Moreover, although *RU* (Mongolian rhubarb) has long been used empirically for the treatment of hepatitis and liver cancer, it has not been studied specifically with respect to its anti-liver cancer activity. Our findings confirm that a lower dose of *RU*-EtOH is required to inhibit HepG2 cell growth than the doses required of the five other solvents investigated in this study. In agreement with the findings reported by Eot [24], *RC*-EtOH did not exhibit a cytotoxic effect on HepG2 cells at an equal dose to that of *RU* extract in this study.

Apoptosis, a programmed cell death that does not affect bordering cells, is considered as a good target for anticancer treatment [30]. We found that the *RU*-EtOH dose-dependently induced HepG2 cell apoptosis through the activation of caspase-3 and cleavage of PARP, resulting in DNA fragmentation. Moreover, the *RU*-EtOH showed the highest ability to chelate iron (data not shown in this study); thus, the apoptotic effect of *RU*-EtOH might be closely related to suppressing iron-dependent enzymes such as topoisomerase and iron-containing ribonucleotide reductase, which are important for DNA synthesis, rather than to increasing reactive oxygen species (ROS) in cancer cells [31,32]. However, the correlation between iron chelators, antioxidants, and ROS is still not completely understood in cancer progression, and the exact mechanism of antioxidants in cancer treatment must be further explored.

Each compound, namely chrysophanol, physcion, and emodin, which were found in the *RU* and *RC* extracts in this study, are already known to have antitumor activity in various cancers as well as a synergistic effect on approved drugs for liver cancer [20,33]. The content of chrysophanol, which exhibits a necrotic effect on liver cancer [20,34], was higher in *RU* and *RC* extracts than the contents of the other two compounds. Notably, the results of the current study confirm that *RU*-EtOH induces apoptosis in liver cancer cells regardless of the chrysophanol concentration. Moreover, the concentration of these three compounds was not associated with their cytotoxic effects. Thus, compared with a single compound, an optimal ratio of pure compounds may exert greater effects on cancer cells. However, the pure compounds in the *RU* and *RC* extracts in this study were not comprehensively defined. Additionally, evidence is lacking to prove that a combination of pure compounds is more effective than a mixture of herbal extracts for the cancer treatment.

In conclusion, this study provides the evidence to support that Mongolian rhubarb is superior to Taiwanese rhubarb in terms of its phytochemical contents and antioxidant activity. However, the phytochemical contents, antioxidant ability, and pure compound concentrations depend on solvent polarity. Moreover, an in vitro study confirmed that *RU*-EtOH exerts an apoptotic effect on HepG2, human liver cancer cell line. Thus, Mongolian rhubarb could be an appropriate source of natural antioxidants and potential therapeutic agent for HCC.

## 4. Materials and Methods

### 4.1. Plant Material Collection

The plant materials used in this study were *Rheum undulatum L*. and *Rumex crispus* L. Dried roots of *RU* were purchased from the traditional herb company “Knight and forest friendship”, Ulaanbaatar, Mongolia, and *RC* roots were collected from a mountain in HshinChu, Taiwan. Both species were identified by professor Ling-Ling Yang, an expert of botany in Taiwan, and the Natural Compound Analysis Core in Taipei Medical University, Taipei, Taiwan. The plant material was dried for 15 days at ambient temperature in a dark and well-ventilated place, and kept in an air-tight, amber glass jar to protect from the light. The voucher specimens had been deposited under the numbers of AF303435, and AF303439 in the Department of Pharmacognosy, School of Pharmacy, College of Pharmacy, and College of Medicine, Taipei Medical University, Taipei, Taiwan.

### 4.2. Plant Extraction

The dried roots of *RU* and *RC* were finely chopped and then soaked in five solvents with different polarities n-Hex, EtOAc, Ac, EtOH, and Water (H_2_O). They were then extracted in Soxhlet apparatus for approximately 8 h. All the extracts were filtered and then the solvents were removed using a vacuum freeze dryer (Biobase Biodustry Co. Ltd. Qindao, China). The concentrated extracts of both *RU* and *RC* were dissolved in dimethyl sulfoxide (DMSO), and 50mg/mL stock solution was prepared and stored at −20 °C. In further experiments, the stock solution was diluted with either culture medium or methanol.

### 4.3. Quantitative Phytochemical Analysis of Extracts

#### 4.3.1. Total Phenolic Content

A Folin–Ciocalteau assay [35] was used to estimate the total phenolic content (TPC). To make a calibration curve (y = 2.6819x + 0.0814; R^2^ = 0.9949), gallic acid (GA) was used with several dilutions (62.5–1000 µg/mL). In each well, 20 µL of the extracts, 80 µL of 7.5% sodium bicarbonate (NaHCO_3_), and 100 µL of the assay solution were added and then incubated for 5 min at 50 °C. The absorbance was recorded at 600 nm by using enzyme-linked immunosorbent assay reader. The TPC was calculated using the formula obtained from the calibration curve of GA. TPCs of extracts were presented in milligrams of GA equivalents (GAE) per gram of extracts.

#### 4.3.2. Total Anthraquinone Content

The protocol of Gritsanapan [36] with slight modification was followed to determine the total anthraquinone content (TAC). The calibration curve of emodin (y = 9.6454x + 0.0188; R^2^ = 0.9998), which served as a reference, was created using five sequential concentrations (6.25–100 µg/mL). Next, 100 µL of test solution mixed with 100 µL of 0.5% magnesium acetate. The reaction absorbance was immediately measured at 515 nm. Finally, TAC was calculated using the calibration curve equation and expressed in milligrams of emodin equivalents (EE) per gram of extracts.

#### 4.3.3. Total Flavonoid Content

An aluminium chloride colorimetric assay was used to estimate the total flavonoid content (TFC) according to the method of Gosh [37]. Each 100 µL of test solution was incubated with 100 µL of 2% aluminum chloride solution at ambient temperature for 10 min. The absorbance of all the extracts was then measured at 368 nm. TFCs were quantified from the standard quercetin curve (y = 3.9886x + 0.0267; R^2^ = 0.9999). The results were expressed in milligrams of quercetin equivalent (QE) per gram of extracts.

### 4.4. Standardization and Quantitative Analysis of Anthraquinone Derivatives from Extract of RU and RC through the Use of High-Performance Liquid Chromatography (HPLC)

HPLC analysis, a method of quality control of extracts, was performed to detect the major compounds of *RU* and *RC* extracts. Chrysophanol, emodin, and physcion were reference compounds, and were purchased from Sigma-Aldrich (St. Louis, MO, USA). The 1 mg/mL compounds or extracts were dissolved in the methanol solution to prepare stock solution, and then a 0.45-μm Millipore filter was used to filter the solution. Then, stock solutions were diluted to obtain 500 μg/mL of extracts and 25 μg/mL of compounds for the HPLC analysis. Testing compounds were analyzed in an HPLC system (Hitachi High-Technologies, Tokyo, Japan) equipped with a 250-nm detector or fluorescence detector (Ex: 430 nm, Em: 525 nm) and LiChroCART RP-C_18_ column (4.6 mm i.d. × 250 mm, 5 μm, Merck, Darmstadt, Germany). The mobile phase was constituted of MeCN-H_2_O (0.05% TFA): 0 min, 55/45; 10 min, 60/40, 15 min, 85/15, 20 min, 85/15; 25 min, 55/45; 30 min, 55/45 and the flow rate was 1.0 mL/min.

### 4.5. Analysis of Antioxidant and Free Radical Scavenging Activities

The percentage of free radical scavenging activity was calculated using the following formula with 2,2′-azinobis-(3-ethylbenzothiazoline-6-sulfonic acid) (ABTS) and 2,2-diphenyl-1-picrylhydrazyl (DPPH) assays. All the results of scavenging activities were presented as the half-maximum inhibition concentration. Trolox and GA were used as a positive control.

(1) $$\mathrm{Scavenging}\;\%=\frac{{\mathrm{Abs}}_{\mathrm{control}}-{\mathrm{Abs}}_{\mathrm{sample}}}{{\mathrm{Abs}}_{\mathrm{control}}}\times 100\%$$

#### 4.5.1. ABTS Scavenging Activity

To prepare the stock solution for ABTS assays [38], the stock solution of ABTS was prepared according to specific protocol, and incubated overnight before every experiment. The ABTS stock solution was then diluted several times to prepare a testing solution with an absorbance of 0.7 to 1.0 for the experiment. GA and Trolox were used as a standard solution and methanol was used as a blank solution. In each well, 100 µL of ABTS testing solution was added and followed by 100 µL of extracts with different concentrations. The absorbance was recorded at 734 nm after 10 min of incubation. The scavenging activity of each extract was calculated using the aforementioned formula.

#### 4.5.2. DPPH Scavenging Activity

DPPH scavenging was carried out using the method developed by Om P.Sharma [39] with slight modification. The stock of extracts was added to 100 µL of 200 µM DPPH and incubated for 30 min in a dark environment at ambient temperature. GA and Trolox were reference compounds. The absorbance was detected at 517 nm through spectrophotometry, and the IC50 dose of extracts was computed in a similar manner to the computation for the ABTS scavenging assay.

#### 4.5.3. Ferric Reducing Antioxidant Power Assay (FRAP)

A FRAP assay was done using the method proposed by Faria, A [40]. Each extract (20 µL) was mixed with 200 µL of FRAP reagent and incubated at ambient temperature for 8 min; the absorbance of the mixture was then recorded at 595 nm. A calibration curve was organized with ferrous sulfate (y = 0.7266x − 0.005; R^2^ = 0.9993), and the results were expressed in millimole ferrous ion equivalents per gram dry weight of the sample (mmol Fe^2+^/g DW).

### 4.6. In Vitro Assays

#### 4.6.1. Cell Culture

HepG2 cell lines were provided by The Cancer Research Laboratory of Wan Fang Hospital, affiliated by Taipei Medical University, Taipei, Taiwan. The cells were grown in a 10-cm dish with Dulbecco’s modified Eagle’s medium supplemented with 10% heat-inactivated fetal bovine serum, 5% penicillin/streptomycin (1000 µg/mL). The cells were passaged every two days.

#### 4.6.2. Cell Viability Assay: (3-(4,5-dimethylthiazol-2-yl)-2,5-diphenyltetrazolium Bromide Assay (MTT Assay)

HepG2 cells were seeded on 96-well plates with 1 × 10^5^cell/well density. Next, the cells were treated with different doses of extracts and incubated for the following time periods: 24, 48, and 72 h. Thereafter, the medium was replaced with 10 µL of a 12 mM MTT solution in 100 µL of medium and kept at 37 °C for 4 h. After incubation, the medium was substituted with 100 µL of DMSO to dissolve the MTT crystals, and then absorbance was measured at 570 nm through spectrophotometry. The cell viability percentage was estimated from a comparison of the absorbances of treated and untreated cells. The results were expressed as IC50 doses.

### 4.7. DNA Fragmentation Assay

HepG2 (8 × 10^5^) were seeded in each well of a six-well plate and incubated overnight. Then, the cells were treated with EtOH extracts of *RU* and *RC* and incubated for 24, 48, and 72 h. Next, scrapping and centrifuging at 2000 rpm for 5 min were performed to collect the cells from each well. After removal of the medium, the samples were washed with phosphate-buffered saline (PBS) once. To each sample, 100 µL of DNA lysis buffer was added and incubated overnight at 61 °C. The next day, 1 µL of RNAse A (10 mg/mL) was added to each well and incubated for 1 h under the previous condition. Subsequently, 100 µL of phenol–chloroform was added and centrifuged at 12,000 rpm for 30 min to isolate and then collect DNA samples.

#### 4.7.1. Gel Preparation

Agarose gel was prepared with a 2% concentration. A microwave was used to dissolve agarose powder in TBE buffer. Agarose solution (20 mL) with 2 µL of nucleic acid staining was placed in a gel tank and kept at ambient temperature for 30 min to solidify.

#### 4.7.2. DNA Loading Procedure

DNA samples (20 µL) were mixed with 3 µL of DNA loading dye. The mixture was then added to the agarose gel placed in the running tank. Samples were run at a voltage of 50 mAh for 40 min. Pictures were captured under ultraviolet light.

### 4.8. Western Blot

HepG2 cells were treated with EtOH extracts of *RU* and *RC* for different time durations and then harvested. Treated cells washed with PBS were lysed in RIPA (Sigma-Aldrich) overnight, and protein samples were collected through centrifugation at 12,000 rpm for 30 min at 4 °C. A calibration curve was made with 1 mg/mL bovine serum albumin (BSA) to estimate the protein concentrations of the samples. The protein samples were mixed with the loading dye in equal proportions and denaturized at 95 °C for 5 min. The prepared proteins were separated using polyacrylamide gels with sodium dodecyl sulfate and transferred to immobilon polyvinylidene difluoride membranes. The membranes were incubated in 1% BSA for 1 h and kept with indicated antibodies at 4 °C overnight. Next, the membranes were added to alkaline phosphatase-conjugated immunoglobulin G antibody for 1 h. Proteins were evaluated using colorimetric substrates of nitro blue tetrazolium and 5-btomo-4-chloro-3-indlyl phosphate.

### 4.9. Annexin V-Fluorescein Isothiocyanate/Propidium Iodide Analysis

To detect apoptosis induced by the EtOH extracts of *RU* and *RC*, an Annexin V-fluorescein isothiocyanate (FITC) apoptosis detection kit (Strong Biotech Corporation, Taipei, Taiwan) was used. Cells were plated on six-well plates with a density of 8 × 10^5^ cells/well. The cells were treated with different doses of EtOH extracts from *RU* and *RC*. Supernatants and cells were collected together and centrifuged at 2000 rpm for 5 min. Samples were washed with PBS twice and then stained with FITC-conjugated Annexin V and propidium iodide for 15 min in a dark environment. The results were evaluated using CytoFlex (Beckman Coulter, Brea, CA, USA).

### 4.10. Statistical Analysis

The quantitative results were expressed as the mean ± standard error of the mean based on triplicate experiments and analyzed by one-way and two-way ANOVA. A *p*-value of < 0.05 was considered statistically significant. GraphPad Prism 8 software (GraphPad, Inc., San Diego, CA, USA) was used to analyze and express the data.

## Figures and Tables

**Table 1:** Phytochemical contents of *RU* and *RC* extracts in different solvents.

	Total Phenols (mg GAE/g DW)		Total Anthraquinones (mg EE/g DW)		Total Flavonoids (mg QE/g DW)	
	*RU*	*RC*	*RU*	*RC*	*RU*	*RC*
n-Hex	2.44 ± 0.69	4.23 ± 0.36 ^ns^	94.10 ± 4.41 ^b^	73.65 ± 11.30	47.47 ± 1.64	192.60 ± 0.47 ^a^
EtOAc	11.16 ± 1.41 ^a^	2.89 ± 0.29	33.68 ± 2.28	34.14 ± 5.47 ^ns^	50.98 ± 1.79	70.99 ± 3.72 ^b^
Ac	8.45 ± 0.61 ^b^	5.01 ± 0.08	15.14 ± 2.47	43.54 ± 3.82 ^a^	37.92 ± 12.44	121.63 ± 10.87 ^a^
EtOH	9.57 ± 1.03 ^a^	2.96 ± 0.17	25.15 ± 2.69 ^b^	11.99 ± 2.90	50.70 ± 2.96 ^ns^	42.66 ± 1.21
H_2_O	8.39 ± 1.83 ^a^	3.5 ± 0.44	9.43 ± 3.31	12.73 ± 2.27 ^ns^	49.63 ± 10.53 ^ns^	39.51 ± 4.96

*RU*: *Rheum undulatum*, *RC*: *Rumex crispus*, GAE: gallic acid equivalent, EE: emodin equivalent, QE: quercetin equivalent, DW: dry weight, n-Hex: n-hexane, EtOAc: Ethyl acetate, Ac: Acetone, EtOH: Ethanol, H_2_O: Water. Value represent mean ± standard error of the mean. Different letters between *RU* and *RC* in each phytochemical content represent significant difference: ^a^ *p* < 0.0001; ^b^ *p* < 0.05; ns: not significant.

**Table 2:** Quantitative HPLC analysis of the *RU* and *RC* extracts.

	*RU*			*RC*		
	Chrysophanol	Physcion	Emodin	Chrysophanol	Physcion	Emodin
n-Hex	120.06 ± 13.82 ^a^	19.02 ± 2.58 ^ns^	3.33 ± 0.54	17.25 ± 5.23	20.34 ± 3.69	10.59 ± 2.47 ^a^
EtOAc	58.83 ± 6.29 ^a^	8.07 ± 0.82 ^ns^	6.51 ± 1.67 ^ns^	5.01 ± 0.27	6.48 ± 1.02	5.19 ± 0.89
Ac	24.15 ± 2.69 ^a^	3.5 ± 0.58	3.12 ± 0.21	6.47 ± 0.59	7.05 ± 1.98 ^a^	5.42 ± 1.02 ^a^
EtOH	28.81 ± 2.08 ^a^	4.53 ± 0.34 ^a^	2.39 ± 0.27 ^a^	0.09 ± 0.02	0.98 ± 0.19	0.74 ± 0.08
H_2_O	1.29 ± 0.2 ^a^	0.79 ± 0.14 ^a^	0.26 ± 0.08 ^a^	0 ± 0.00	0.40 ± 0.06	0 ± 0.01

(Concentrations = mg compound/g extract) HPLC: high-performance liquid chromatography, *RU*: *Rheum undulatum*, *RC*: *Rumex crispus*, n-Hex: n-hexane, EtOAc: Ethyl acetate, Ac: Acetone, EtOH: Ethanol, H_2_O: Water. Data represent mean ± standard error of the mean. Different letters between *RU* and *RC* in each solvent extract represent significant difference: ^a^ *p* < 0.05; ns: not significant.

**Table 3:** Antioxidant and free radical scavenging activities of *RU* and *RC* extracts.

	IC_50_ (µg/mL)				FRAP (mMFe^2+^/g)	
	ABTS		DPPH			
GA	3.16 ± 0.15		1.25 ± 0.20		nt	
Trolox	13.53 ± 0.40		6.28 ± 1.58		nt	
	*RU*	*RC*	*RU*	*RC*	*RU*	*RC*
n-Hex	93.23 ± 4.18	75.12 ± 8.98 ^a^	1800.87 ± 151.03	74.39 ± 0.01 ^a^	0.99 ± 0.30	1.79 ± 0.13 ^b^
EtOAc	5.67 ± 0.23 ^a^	207.17 ± 2.50	41.37 ± 1.98 ^b^	246.76 ± 20.02	6.42 ± 0.24 ^a^	1.26 ± 0.15
Ac	10.25 ± 0.51 ^a^	52.05 ± 2.64	53.49 ± 4.93 ^ns^	70.01 ± 0.01	5.17 ± 0.19 ^a^	2.65 ± 0.13
EtOH	14.08 ± 0.13 ^a^	77.95 ± 3.31	52.12 ± 3.29 ^ns^	146.72 ± 1.46	4.66 ± 0.04 ^a^	1.98 ± 0.17
H_2_O	14.09 ± 1.88 ^a^	70.55 ± 1.81	46.15 ± 4.66 ^ns^	137.40 ± 1.82	4.86 ± 0.26 ^a^	2.34 ± 0.22

*RU*: *Rheum undulatum*, *RC*: *Rumex crispus*, ABTS: 2,2′-azinobis-(3-ethylbenzothiazoline-6-sulfonic acid), DPPH: 2,2-diphenyl-1-picrylhydrazyl, FRAP: ferric reducing antioxidant power, n-Hex: n-hexane, EtOAc: ethyl acetate, Ac: acetone, EtOH: ethanol, H_2_O: water, nt: not tested. Data represent mean ± standard error of the mean. Different letters between *RU* and *RC* in each assay represent significant difference: ^a^ *p* < 0.0001; ^b^ *p* < 0.05; ns: not significant.

**Table 4:** IC50 values after 24, 48, and 72 h of treatment incubation of *RU* and *RC* extracts.

Solvents	*RU* (IC50 µg/mL)			*RC* (IC50 µg/mL)		
	24 h	48 h	72 h	24 h	48 h	72 h
n-Hex	625.22 ± 30.73	419.77 ± 13.20	427.55 ± 21.54	nt	1973.14 ± 257.15	146.09 ± 24.99
EtOAc	449.82 ± 51.68	208.46 ± 30.63	191.63 ± 26.31	nt	925.30 ± 126.58	468.95 ± 7.59
Ac	403.69 ± 52.45	187.18 ± 20.59	170.16 ± 28.03	nt	606.56 ± 41.88	456.74 ± 20.10
EtOH	262.28 ± 13.99	171.94 ± 6.56	167.09 ± 8.99	2792.33 ± 396.28	1753.02 ± 77.84	1617.32 ± 31.49
H2O	880.33 ± 64.16	555.84 ± 2.09	635.09 ± 22.58	2465.74 ± 317.40	1397.90 ± 30.76	1176.79 ± 16.36

*RU*: *Rheum undulatum*, *RC*: *Rumex crispus*, n-Hex: n-hexane, EtOAc: ethyl acetate, Ac: acetone, EtOH: ethanol, H_2_O: water, nt: not tested. Data represent mean ± standard error of the mean.

## Data Availability

The data are available in this article and raw data is available upon request to the author G.J.

## Sample Availability

Not available.
